# CA125 outperforms NT-proBNP in the prediction of maximum aerobic capacity in heart failure with preserved ejection fraction and kidney dysfunction

**DOI:** 10.1093/ckj/sfae199

**Published:** 2024-07-02

**Authors:** Gonzalo Núñez-Marín, Patricia Palau, Eloy Domínguez, Rafael de la Espriella, Laura López, Cristina Flor, Paloma Marín, Miguel Lorenzo, Gema Miñana, Vicent Bodí, Juan Sanchis, Julio Núñez

**Affiliations:** Department of Cardiology, Hospital Clínico Universitario de Valencia, Valencia, Spain; Department of Cardiology, Hospital Clínico Universitario de Valencia, Valencia, Spain; Faculty of Medicine, Universitat de València, Valencia, Spain; Department of Cardiology, Hospital Clínico Universitario de Valencia, Valencia, Spain; Department of Cardiology, Hospital Clínico Universitario de Valencia, Valencia, Spain; Centro de Investigación Biomédica en Red de Enfermedades Cardiovasculares, Madrid, Spain; Faculty of Physiotherapy, Universitat de València, Valencia, Spain; Faculty of Physiotherapy, Universitat de València, Valencia, Spain; Department of Cardiology, Hospital Clínico Universitario de Valencia, Valencia, Spain; Department of Cardiology, Hospital Clínico Universitario de Valencia, Valencia, Spain; Department of Cardiology, Hospital Clínico Universitario de Valencia, Valencia, Spain; Department of Cardiology, Hospital Clínico Universitario de Valencia, Valencia, Spain; Faculty of Medicine, Universitat de València, Valencia, Spain; Centro de Investigación Biomédica en Red de Enfermedades Cardiovasculares, Madrid, Spain; Department of Cardiology, Hospital Clínico Universitario de Valencia, Valencia, Spain; Faculty of Medicine, Universitat de València, Valencia, Spain; Centro de Investigación Biomédica en Red de Enfermedades Cardiovasculares, Madrid, Spain; Department of Cardiology, Hospital Clínico Universitario de Valencia, Valencia, Spain; Faculty of Medicine, Universitat de València, Valencia, Spain; Centro de Investigación Biomédica en Red de Enfermedades Cardiovasculares, Madrid, Spain

**Keywords:** CA125, cardiorenal syndrome, HFpEF, maximal aerobic capacity, natriuretic peptides

## Abstract

**Background:**

Heart failure with preserved ejection fraction (HFpEF) often coexists with chronic kidney disease (CKD). Exercise intolerance is a major determinant of quality of life and morbidity in both scenarios. We aimed to evaluate the associations between N-terminal pro-B-type natriuretic peptide (NT-proBNP) and carbohydrate antigen 125 (CA125) with maximal aerobic capacity (peak VO_2_) in ambulatory HFpEF and whether these associations were influenced by kidney function.

**Methods:**

This single-centre study prospectively enrolled 133 patients with HFpEF who performed maximal cardiopulmonary exercise testing. Patients were stratified across estimated glomerular filtration rate (eGFR) categories (<60 ml/min/1.73 m^2^ versus ≥60 ml/min/1.73 m^2^).

**Results:**

The mean age of the sample was 73.2 ± 10.5 years and 56.4% were female. The median of peak VO_2_ was 11.0 ml/kg/min (interquartile range 9.0–13.0). A total of 67 (50.4%) patients had an eGFR <60 ml/min/1.73 m^2^. Those patients had higher levels of NT-proBNP and lower peak VO_2_, without differences in CA125. In the whole sample, NT-proBNP and CA125 were inversely correlated with peak VO_2_ (*r* = −0.43, *P* < .001 and *r* = −0.22, *P* = .010, respectively). After multivariate analysis, we found a differential association between NT-proBNP and peak VO_2_ across eGFR strata (*P* for interaction = .045). In patients with an eGFR ≥60 ml/min/1.73 m^2^, higher NT-proBNP identified patients with poorer maximal functional capacity. In individuals with eGFR <60 ml/min/1.73 m^2^, NT-proBNP was not significantly associated with peak VO_2_ [β = 0.02 (95% confidence interval −0.19–0.23), *P* = .834]. Higher CA125 was linear and significantly associated with worse functional capacity without evidence of heterogeneity across eGFR strata (*P* for interaction = .620).

**Conclusions:**

In patients with stable HFpEF, NT-proBNP was not associated with maximal functional capacity when CKD was present. CA125 emerged as a useful biomarker for estimating effort intolerance in HFpEF irrespective of the presence of CKD.

## Graphical Abstract

**Graphical Abstract:**
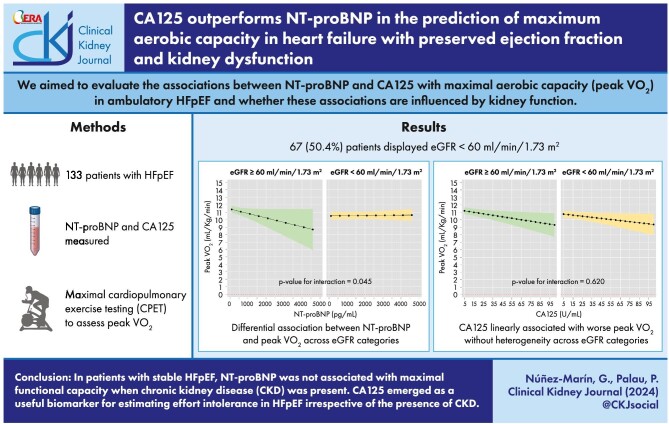

KEY LEARNING POINTS **What was known:**

- Heart failure with preserved ejection fraction (HFpEF) often coexists with chronic kidney disease (CKD) and exercise intolerance is a major determinant of quality of life and morbidity in both scenarios.
- The value of N-terminal pro-B-type natriuretic peptide (NT-proBNP) and carbohydrate antigen 125 (CA125) for estimating maximal functional capacity in HFpEF is not well known, especially in those in which CKD coexists.

**This study adds:**

- NT-proBNP is not useful for assessing maximal aerobic capacity assessed by maximal aerobic capacity (peak VO_2_) in patients with HFpEF and significant kidney dysfunction (CKD stage ≥3).
- Higher CA125 levels were linearly associated with worse functional capacity, regardless of the presence of CKD.

**Potential impact:**

- NT-proBNP should not be considered a useful parameter for assessing peak VO_2_ in patients with HFpEF and significant kidney dysfunction.
- Alternatively, in patients with HFpEF with and without cardiorenal syndrome, CA125 may be a useful circulating biomarker for estimating functional capacity.

## INTRODUCTION

Heart failure with preserved ejection fraction (HFpEF) is a multifactorial syndrome with high morbimortality burden [1–3]. It is highly prevalent in women, elderly people and patients with multiple comorbidities, including chronic kidney dysfunction [3–5]. In HFpEF, exercise intolerance is a major determinant of quality of life and morbidity [6–9]. Similarly, physical performance and aerobic exercise capacity are impaired in chronic kidney disease (CKD) and are associated with increased mortality and reduced quality of life [10, 11]. The mechanisms behind this poor physical condition may be shared in HFpEF and CKD and involve endothelial dysfunction, low cardiovascular reserve, inflammation and sarcopenia [12, 13]. Peak exercise oxygen uptake (peak VO_2_) assessed by cardiopulmonary exercise testing (CPET) represents an accurate parameter to measure maximal aerobic capacity in both scenarios. Nonetheless, CPET is a time-consuming exam that only a minority of patients may access in real clinical practice. Accordingly, defining widely available surrogate parameters that accurately predict poor functional capacity is a pertinent goal. In this sense, higher natriuretic peptides (NPs) have been shown to be associated with poor effort capacity, mainly in heart failure with reduced ejection fraction (HFrEF) [14–16]. The value of N-terminal pro-B-type natriuretic peptide (NT-proBNP) for estimating peak VO_2_ in HFpEF is not well known, especially in those in which CKD coexists, a common condition in which we find higher-than-expected values of NPs [17]. Conversely, carbohydrate antigen 125 (CA125) has been shown to be a reliable proxy of fluid overload, inflammation and clinical course in heart failure (HF), regardless of left ventricular ejection fraction (LVEF) [18, 19]. Interestingly, CA125, as a high-molecular protein, is not significantly influenced by renal function [17, 18]. This characteristic renders it a potentially valuable biomarker in cardiorenal syndromes.

In this work, we aimed to evaluate the associations between NT-proBNP and CA125 with peak VO_2_ in ambulatory HFpEF and whether these associations are influenced by kidney function.

## MATERIALS AND METHODS

### Study design and population

This study prospectively enrolled 156 consecutive patients with HFpEF in a single third-level centre in Spain. All study participants were previously followed up in a specialized HF clinic. Subjects were eligible if they were >18 years of age; able to provide written informed consent; had a previous history of symptomatic HF [New York Heart Association (NYHA) functional class II–III]; had a normal LVEF (ejection fraction ≥50% by the Simpson method); had objective evidence of cardiac structural, functional and serological abnormalities consistent with the presence of left ventricular diastolic dysfunction or elevated left ventricular filling pressures (echocardiography at rest with relative wall thickness >0.42; or left atrial volume >34 ml/m^2^ or >40 ml/m^2^ in atrial fibrillation; or *E*:*e*′ ratio >9 or tricuspid regurgitation velocity >2.8 m/s; or NT-proBNP >125 pg/ml in sinus rhythm or >365 pg/ml in atrial fibrillation) and were stable (defined as no hospital admission in the past 3 months) [1]. Exclusion criteria consisted of life expectancy of <1 year, moderate or severe valvular heart disease, previously diagnosed amyloid or restrictive cardiomyopathies, history of pulmonary disease (based on the previous medical history of the patient that was extracted from the electronic medical record and including known pulmonary arterial hypertension, chronic thromboembolic pulmonary disease or more than moderate chronic obstructive pulmonary disease defined by forced expiratory volume <50%), known active cancer except for non-melanoma skin cancers and impossibility to perform a valid CPET. The protocol was approved by the research ethics committee following the principles of the Declaration of Helsinki and local regulations. All patients provided written informed consent. To ensure this observational study's comprehensive and transparent reporting, we have adhered to the guidelines outlined in the Strengthening the Reporting of Observational Studies in Epidemiology (STROBE) checklist. The completed STROBE checklist has been included as Supplementary Table S1.

### Procedures

Patients underwent a maximal symptom-limited CPET on a bicycle ergometer using a ramp protocol of 10 W increments every 1 minute. Maximal functional capacity was defined as when the patient stops pedalling because of symptoms, and the respiratory exchange ratio (RER) was ≥1.05. Peak VO_2_ <12 ml/min/kg was the criteria for defining severe functional impairment according to a consensus document [20]. The heart rate (HR) was evaluated at rest (rest HR) and peak effort (peak HR). The HR response during CPET was evaluated following the chronotropic index formula: peak HR − rest HR/[(220 − -age) – rest HR)] [21, 22].

The same day of CPET, all patients underwent a complete clinical examination, physician-perceived NYHA class, resting echocardiography in accordance with published guidelines using standard views and techniques [23] and laboratory tests (including a complete blood count, serum electrolytes, blood urea nitrogen, serum creatinine, NT-proBNP and CA125). Estimated glomerular filtration rate (eGFR) was calculated using the Chronic Kidney Disease Epidemiology Collaboration equation using demographics and serum creatinine [24]. Plasma concentrations of NT-proBNP and CA125 were measured using the commercially available electrochemiluminescent sandwich immunoassays (Roche Elecsys NT-proBNP assay and Roche Elecsys CA 125 assay; Roche, Basel, Switzerland).

### Statistical analysis

Continuous variables were expressed as mean [± standard deviation (SD)] or median [interquartile range (IQR)], as appropriate. Discrete variables were summarized as percentages. Baseline characteristics across eGFR categories (<60 ml/min/1.73 m^2^ versus ≥60 ml/min/1.73 m^2^) were compared using the *t*-test (parametric distribution) or Wilcoxon test (non-parametric distribution) for continuous variables and the χ^2^ test for categorical variables. The Spearman correlation index was used to test the correlations between both exposures and peak VO_2_. Uni- and multivariate regression models were performed to test the independent association between both biomarkers (NT-proBNP and CA125) and peak VO_2_ across eGFR status. Covariates included in the multivariate analysis were age, sex, haemoglobin, body mass index (BMI), Charlson comorbidity index, atrial fibrillation, chronotropic index, RER, LVEF, indexed left ventricular volume and *E*:*e*′ ratio. The use of CA125 and NT-proBNP for diagnosis of peak VO_2_ <12 ml/min/kg was evaluated and compared by the area under the receiver operating characteristics curves (AUC). Stata 18.0 (StataCorp, College Station, TX, USA) was used for the analyses. The full model explained 64.5% of the variability of peak VO_2_ (adjusted *R*^2^).

## RESULTS

### Baseline characteristics

**Figure 1:**
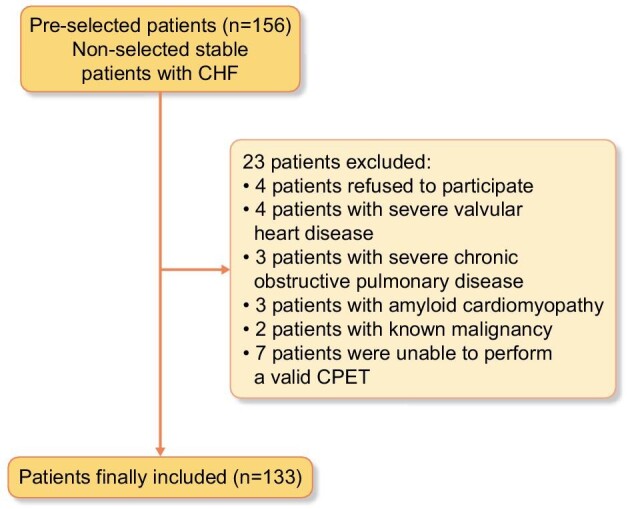
Flow chart of the protocol used for the enrolment of patients in this study. CHF: chronic heart failure.

Of the 156 initially screened patients, 23 were excluded because they met one of the exclusion criteria. Thus 133 patients were finally included. Figure 1 illustrates the protocol used for enrolling patients in this study. The mean age of the sample was 73.2 ± 10.5 years, 56.4% were female, 33.8% were in NYHA class III and 68 (51.1%) had atrial fibrillation. Most patients were on loop diuretics (84.2%), beta-blockers (88.7%) or angiotensin-converting enzyme inhibitors or angiotensin receptor blockers (68.4%). The median eGFR, NT-proBNP and CA125 were 58.4 ml/min/1.73 m^2^ (IQR 43.6–74.2), 556.0 pg/ml (IQR 288.0–1399.0) and 12.0 U/ml (IQR 8.0–19.0), respectively. A total of 67 (50.4%) patients had an eGFR <60 ml/min/1.73 m^2^. The median peak VO_2_ was 11.0 ml/kg/min (IQR 9.0–13.0) and 58.6% of patients had a peak VO_2_ <12 ml/kg/min. Baseline characteristics in the whole sample and across eGFR strata are summarized in Table 1. Overall, the group of patients with eGFR <60 ml/min/1.73 m^2^ were older, with a higher proportion of males, more prevalence of ischaemic heart disease and a higher Charlson comorbidity index when compared with patients with an eGFR ≥60 mL/min/1.73 m^2^. They also had lower haemoglobin and higher levels of NT-proBNP, without differences in CA125, and lower peak VO_2_.

**Table 1:** Baseline characteristics of the population stratified across eGFR status.

Variables	All patients (*N* = 133)	eGFR ≥60 ml/min/1.73 m^2^ (*n* = 66)	eGFR <60 ml/min/1.73 m^2^ (*n* = 67)	*P*-value
Demographic and medical history				
Age (years), mean ± SD	73.2 ± 10.5	70.3 ± 12.1	76.1 ± 7.5	.001
Female, *n* (%)	75 (56.4)	43 (65.2)	32 (47.8)	.043
Hypertension, *n* (%)	120 (90.2)	60 (90.9)	60 (89.6)	.792
Dyslipidaemia, *n* (%)	102 (76.7)	51 (77.3)	51 (76.1)	.609
Diabetes, *n* (%)	27 (40.9)	27 (40.9)	32 (47.8)	.426
BMI (kg/m^2^), median (IQR)	31.1 (28.0–34.3)	31.4 (28.2–34.2)	31.0 (27.4–34.3)	.612
Smoker, *n* (%)	6 (4.5)	2 (3.0)	4 (6.0)	.414
Former smoker, *n* (%)	17 (25.8)	24 (35.8)	41 (30.8)	.209
Ischaemic heart disease, *n* (%)	41 (30.8)	15 (22.7)	26 (38.8)	.045
Atrial fibrillation, *n* (%)	68 (51.1)	33 (50.0)	35 (52.2)	.796
History of stroke, *n* (%)	9 (6.8)	4 (6.1)	5 (7.5)	.748
NYHA class III, *n* (%)	45 (33.8)	20 (30.3)	25 (37.3)	.393
Charlson comorbidity index, median (IQR)	2 (1–3)	2 (1–2)	3 (2–4)	<.001
Medical treatment, *n* (%)				
ACEi/ARB	91 (68.4)	49 (74.2)	42 (62.7)	.152
MRA	29 (21.8)	10 (15.2)	19 (28.4)	.065
Betablockers	118 (88.7)	61 (92.4)	57 (85.1)	.180
Loop diuretics	112 (84.2)	56 (84.8)	56 (83.6)	.841
Echocardiographic parameters, median (IQR)				
LVEF (%)	67.0 (60.0–71.8)	67.0 (60.0–71.8)	66.0 (60.0–74.0)	.737
LVEDV (ml/m^2^)	45.7 (37.8–56.1)	44.5 (33.3–53.8)	46.1 (39.9–63.6)	.036
LVESV (ml/m^2^)	14.9 (11.1–18.8)	14.5 (9.1–17.7)	15.2 (13.6–19.8)	.012
Left atrial volume (ml)	80.0 (70.0–90.2)	80.0 (69.0–82.0)	80.0 (72.8–100.0)	.057
CPET parameters, median (IQR)				
HR at rest (bpm)	67.0 (59.0–74.0)	67.5 (59.0–74.0)	66.0 (59.0–74.0)	.787
Peak VO_2_ (ml/kg/min)	11.0 (9.0–13.0)	12.0 (9.7–13.0)	10.0 (8.0–12.0)	.001
pp-peak VO_2_ (%)	64.1 (53.0–74.4)	67.6 (55.4–78.9)	60.3 (47.0–70.9)	.009
VE/VCO_2_ slope	34.7 (31.0–38.9)	32.4 (27.9–36.0)	36.5 (32.0–39.9)	<.001
RER	1.1 (1.0–1.2)	1.1 (1.0–1.2)	1.1 (1.0–1.1)	.147
Chronotropic index	0.4 (0.3–0.5)	0.4 (0.3–0.5)	0.4 (0.2–0.6)	.737
Laboratory, median (IQR)				
Haemoglobin (mg/dl)	13.0 (11.7–14.1)	13.3 (12.5–14.4)	12.5 (11.6–13.7)	.012
eGFR (ml/min/1.73 m^2^)	58.4 (43.6–74.2)	74.4 (68.3–84.8)	43.6 (33.5–49.6)	<.001
Sodium (eEq/l)	141.0 (139.0–142.0)	140.5 (139.0–142.0)	141.0 (139.0–142.0)	.326
NT-proBNP (pg/ml)	556.0 (288.0–1399.0)	353.5 (192.0–991.0)	1090.0 (436.0–2229.0)	.001
CA125 (U/ml)	12.0 (8.0–19.0)	11.5 (8.0–21.0)	14.0 (8.0–18.0)	.534

Data are expressed as No. (%), continuous variables as mean ± 1 standard deviation, or medians (interquartile range [IQR]), and discrete variables as frequencies and percentages.

ACEi: angiotensin-converting enzyme inhibitor; AF: atrial fibrillation; ARB: angiotensin receptor blocker; BMI: body mass index; bpm: beats per minute; HR: heart rate; LVEDV: left ventricular end-diastolic volume; LVESV: left ventricular end-systolic volume; MRA: mineralocorticoid receptor antagonist; pp-peak VO_2_: percent of predicted peak VO_2_; VE/VCO_2_ slope: ventilatory efficiency.

### Factors associated with peak VO_2_ in HFpEF: the role of NT-proBNP and CA125 across eGFRs

In the whole sample, NT-proBNP and CA125 were significantly and inversely correlated with peak VO_2_ (*r* = −0.43, *P* < .001 and *r* = −0.22, *P* = .010, respectively). Likewise, NT-proBNP was inversely correlated with eGFR (*r* = −0.42, *P* < .001). CA125 was not correlated with eGFR (*r* = 0.12, *P* = .121).

**Figure 2:**
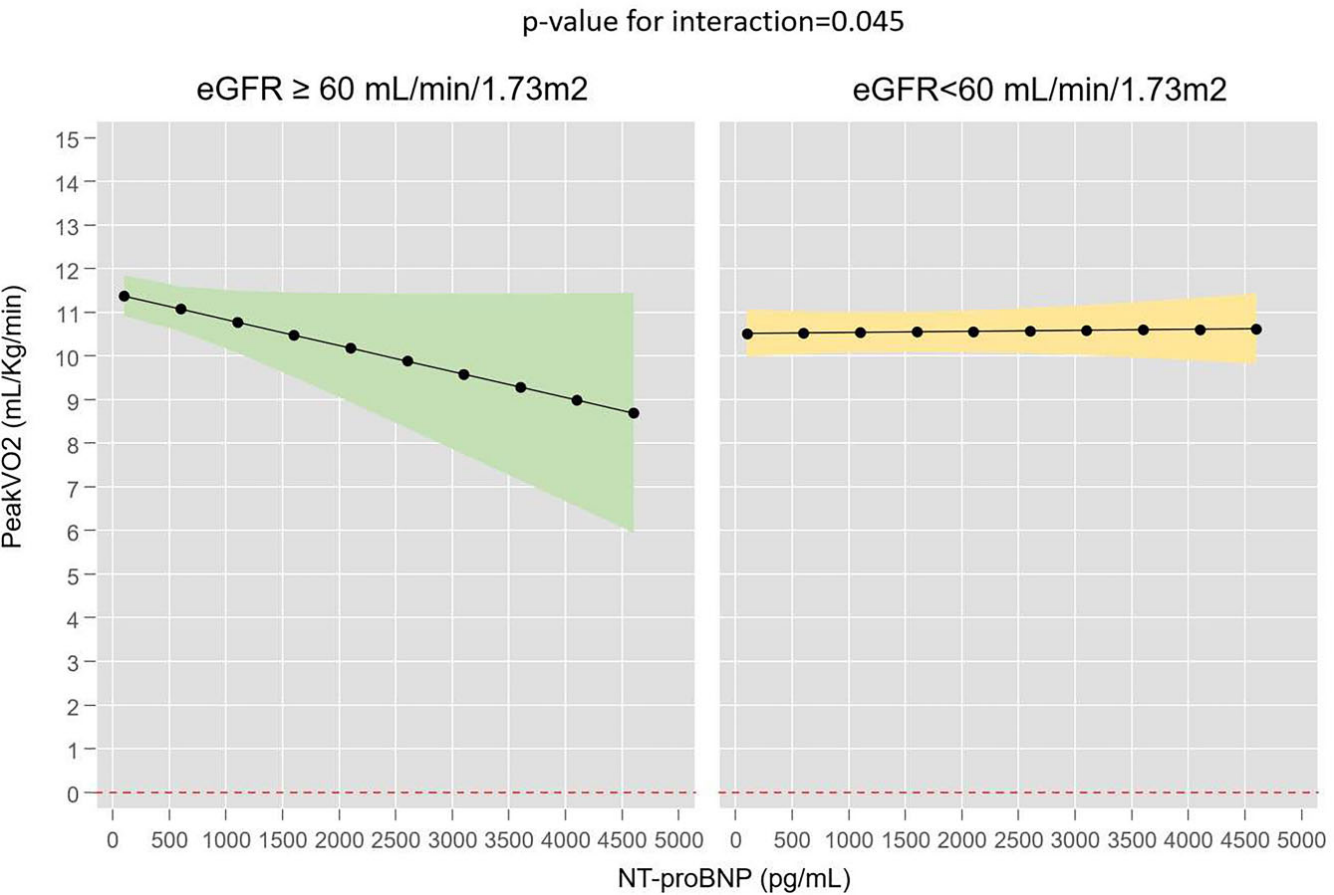
Association between NT-proBNP levels and peak VO_2_ across eGFR ≥60 ml/min/1.73 m^2^ versus <60 ml/min/1.73 m^2^.

The associations (uni- and multivariate) between biomarkers (NT-proBNP and CA125) and peak VO_2_ across eGFR status are presented in Supplementary Table S2. After a full multivariate adjustment, we found a differential association between NT-proBNP and peak VO_2_ across eGFR <60 ml/min/1.73 m^2^ versus ≥60 ml/min/1.73 m^2^ (*P* for interaction = .045). In those patients with eGFR ≥60 ml/min/1.73 m^2^, higher NT-proBNP identified patients at higher risk of poorer maximal functional capacity. In fact, per increase of 1000 pg/ml of NT-proBNP, peak VO_2_ decreased 0.59 ml/kg/min [95% confidence interval (95% CI) −1.23 to −0.04, *P* = .042], as shown in Fig. 2a. Conversely, in individuals with an eGFR <60 ml/min/1.73 m^2^, NT-proBNP was not significantly associated with peak VO_2_ [β = 0.02 ml/kg/min (95% CI −0.19–0.23), *P* = .834], as is shown in Fig. 2b.

**Figure 3:**
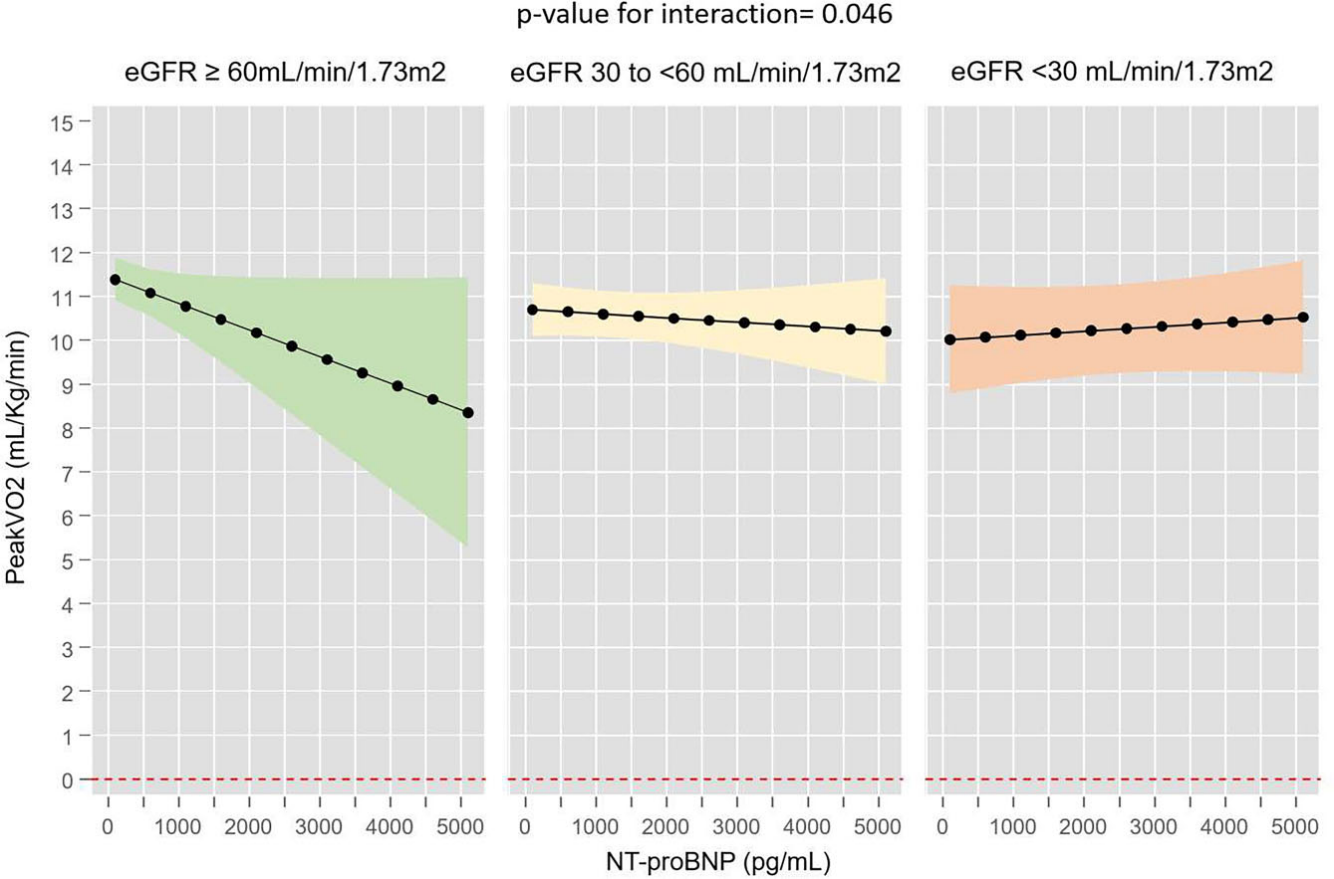
Association between NT-proBNP levels and peak VO_2_ across eGFR ≥60, 30–<60 and <30 ml/min/1.73 m^2^.

These heterogeneous findings were also seen when eGFR was categorized into three categories [<30, 30–<60 and ≥60 ml/min/1.73 m^2^ (*P* for interaction = .046)]. NT-proBNP showed an inverse and linear association with peak VO_2_ in those with an eGFR ≥60 ml/min/1.73 m^2^, but not for patients with an eGFR of 30–<60 or <30 ml/min/1.73 m^2^ (Fig. 3).

**Figure 4:**
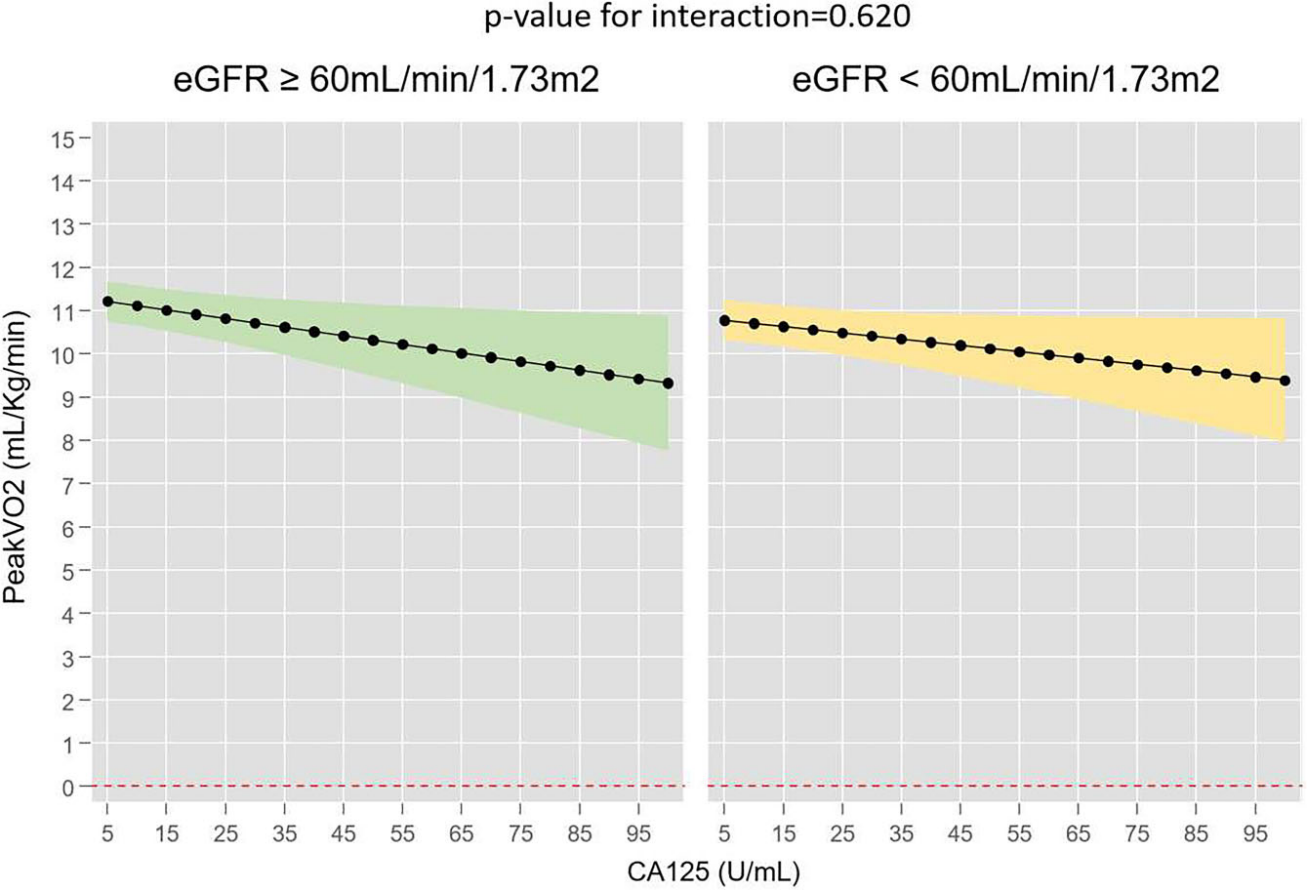
Association between CA125 levels and peak VO_2_ across eGFR ≥60 ml/min/1.73 m^2^ versus <60 ml/min/1.73 m^2^.

**Figure 5:**
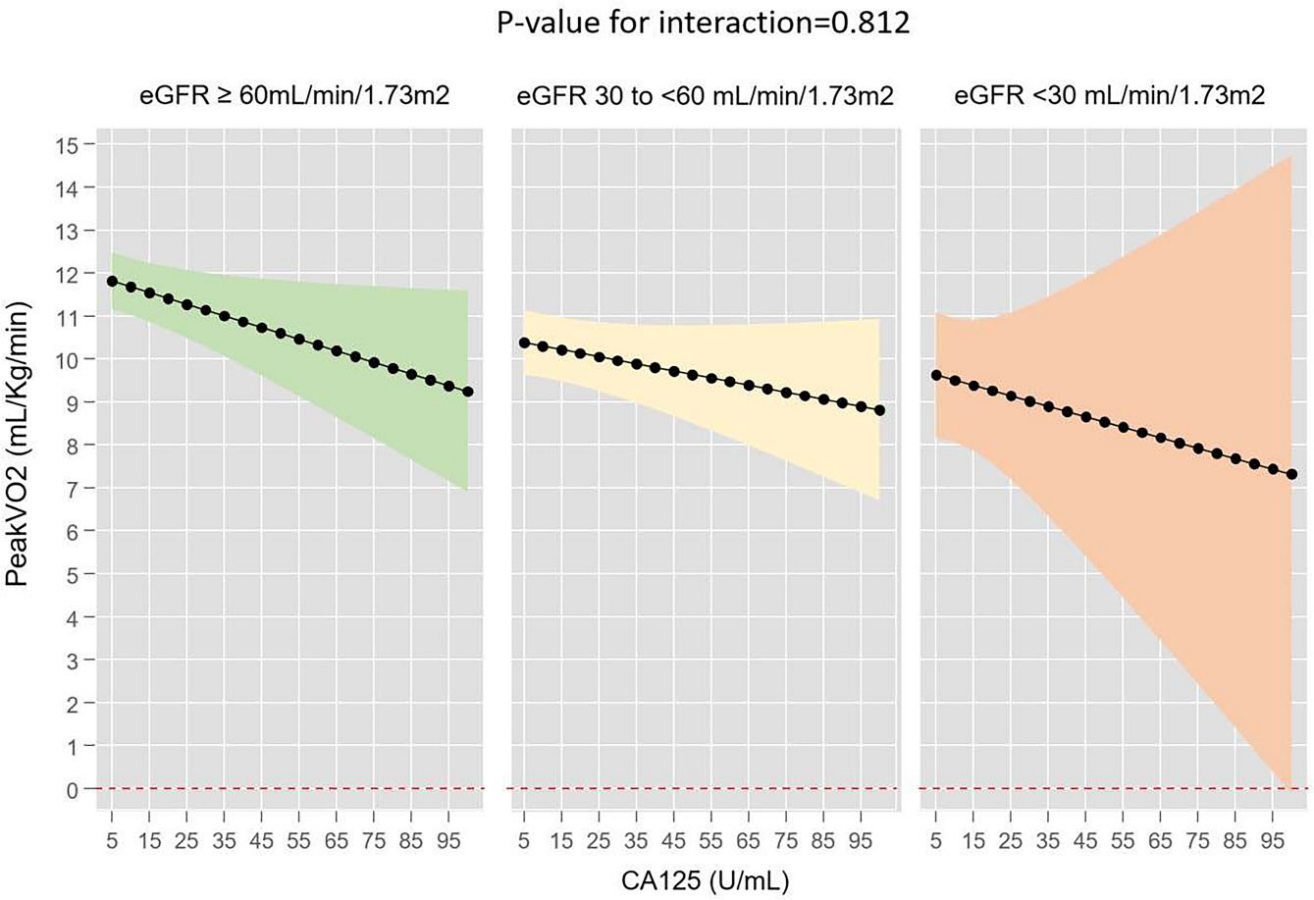
Association between CA125 levels and peak VO^2^ across eGFR ≥60, 30–<60 and <30 ml/min/1.73 m^2^.

Under the same full multivariate setting, including NT-proBNP as a covariate, CA125 emerged as an independent parameter associated with peak VO_2_ regardless of eGFR. In both groups, higher CA125 was linearly associated with worse functional capacity without evidence of heterogeneity (*P* for interaction = .620), as shown in Fig. 4. Thus, per increase of 10 U/ml in CA125, peak VO_2_ decreased by −0.18 ml/kg/min (95% CI −0.34 to −0.02, *P* = .024) when eGFR was <60 ml/min/1.73 m^2^. Similarly, an increase of 10 U/ml in CA125 translated into a reduction in peak VO_2_ of −0.19 ml/kg/min (95% CI −0.35 to −0.02, *P* = .031) in patients with an eGFR ≥60 ml/min/1.73 m^2^. Non-heterogeneous associations between CA125 and peak VO_2_ were also found when eGFR was categorized into three categories (Fig. 5).

When categorizing both biomarkers by their median value, patients with NT-proBNP above the median (>556 pg/ml) had a higher risk of lower peak VO_2_ in eGFR ≥60 ml/min/1.73 m^2^ [β = −1.14 (95% CI −2.25 to −0.30), *P* = .044] but not in eGFR <60 ml/min/1.73 m^2^ [β = −0.71 (95% CI −1.76–0.34), *P* = .185]. Patients with CA125 above the median (>12 U/ml) showed a higher risk of lower peak VO_2_ in eGFR ≥60 ml/min/1.73 m^2^ [β = −0.97 (95% CI −1.97 to −0.02), *P* = .042] and <60 ml/min/1.73 m^2^ [β = −0.82 (95% CI −1.87 to −0.01), *P* = .048].

### Discriminative ability of NT-proBNP and CA125 in identifying peak VO_2_ <12 ml/min/kg

Patients with peak VO_2_ <12 ml/min/kg showed higher NT-proBNP [1095 pg/ml (IQR 441–2016) versus 315 pg/ml (IQR 192–742), *P* < .001] and CA125 values [14 U/ml (IQR 9–21) versus 10 U/ml (IQR 7–17), *P* = .041]. In the overall cohort, the AUC of NT-proBNP versus CA125 for identifying peak VO_2_ <12 ml/min/kg did not differ significantly (0.73 versus 0.67, respectively; *P* = .224). We found a numerical signal indicating that the AUC of NT-proBNP was higher in eGFR ≥60 ml/min/1.73 m^2^ (0.69 versus 0.75 for eGFR <60 ml/min/1.73 m^2^ and eGFR ≥60 ml/min/1.73 m^2^, respectively). For CA125, the AUC was similar across GFR status (0.64 versus 0.70 for eGFR <60 ml/min/1.73 m^2^ and eGFR ≥60 ml/min/1.73 m^2^, respectively).

## DISCUSSION

In a cohort of elderly HFpEF patients with a severely limited functional capacity, we found that NT-proBNP was not a useful parameter for assessing maximal aerobic capacity when significant kidney dysfunction (CKD stage ≥3) was present. Alternatively, in this same population, CA125 emerged as a useful circulating biomarker for estimating functional capacity regardless of the presence of CKD. These findings were supported by evaluating the maximal functional capacity along its continuum or dichotomized.

### NT-proBNP and aerobic capacity in HFpEF according to eGFR strata

The clinical utility of NPs in HF is doubtless. They are useful for diagnosis, risk stratification and monitoring the course of the disease [1, 25]. However, their usefulness is more solid in patients with HFrEF rather than HFpEF, a clinical scenario in which NPs exhibit some diagnostic and prognostic limitations [19, 26, 27]. CKD, a highly prevalent comorbidity in HFpEF, is one of the several reasons contributing to those limitations [27, 28]. Kidney dysfunction is associated with higher circulating levels of NPs due to increased production and impaired clearance, particularly when eGFR is <30 ml/min/1.73 m^2^ [27, 29, 30]. Thus its utility in cardiorenal syndrome may be weaker than in patients with normal kidney function [17]. Regarding their usefulness for assessing functional capacity, several gaps remain. Prior observations in patients with HFrEF from the HF-ACTION trial (NCT00047437) showed that NT-proBNP was a strong predictor of peak VO_2_ after adjustment for 35 demographic and clinical candidate variables [16]. However, although a subanalysis of this trial showed that reduced renal filtration function was associated with markedly impaired cardiorespiratory fitness, a formal interaction between eGFR and NPs in peak VO_2_ prediction was not explored [31]. In HFpEF, only one prior study has reported a very modest association between NPs and peak VO_2_, aligning with previous findings showing that NT-proBNP is a poor surrogate of quality of life in this scenario [32, 33]. To the best of our knowledge, our study is the first one to report a statistically significant differential association between NT-proBNP levels and eGFR categories in the prediction of peak VO_2_ in HFpEF. We found that higher NP levels identified patients with lower peak VO_2_ in patients with an eGFR ≥60 ml/min/1.73 m^2^. In contrast, NT-proBNP was not associated with peak VO_2_ in patients with CKD stage ≥3.

### CA125: a useful biomarker in the cardiorenal patient?

CA125 is a large transmembrane glycoprotein produced in the serosal surfaces such as the pericardium, peritoneum and pleura [18]. Its production is upregulated in response to inflammation, mechanical stress and volume overload [34–36]. Interestingly, CA125 is not substantially modified by LVEF, renal function, age or obesity [19]. Upregulation of CA125 is highly associated with proxies of right-sided heart failure and fluid overload [19]. As such, in acute HF this biomarker provides additional prognostic information beyond NPs and is useful for monitoring in the months–weeks after a decompensation, and two clinical trials found this biomarker useful for tailoring diuretic strategies [37–40]. In HFpEF, higher CA125 levels have consistently been shown to be associated with greater morbimortality burden [38]. In cardiorenal syndrome patients, the evidence is growing. For instance, in acute HF, higher CA125 may help clinicians identify patients with discontinuous intrarenal venous Doppler patterns and select patients to be treated with a more intensive diuretic strategy [37, 41]. Also, a recent large study in acute HF patients (*n* = 4595) reported that the prognostic ability on NT-proBNP for predicting death largely declined at greater CKD severity. Conversely, CA125 remained, showing an independent association with mortality regardless of CKD stage [17]. In the current study, CA125 levels were independently and inversely associated with peak VO_2_ in the whole sample and across eGFR strata. Taken together, these findings confirm the limited value of NPs but expand the utility of CA125 in patients with cardiorenal syndrome. Specifically, we postulate that in patients with stable HFpEF and CKD, higher CA125 levels identify a cluster of patients with poorer aerobic capacity.

### Study limitations and strengths

This study has the strengths of a prospective design and a fair representation of both female (accounting for 56.4% of the sample) and older patients. Also, a good characterization of patients was done, with careful documentation of baseline characteristics, the performance of a maximal symptom-limited CPET on a bicycle ergometer to assess maximal functional capacity and the measurement of both NT-proBNP and CA125 on the same day of CPET using reliable assays. Furthermore, our multivariate analysis was adjusted for well-known prognostic factors.

Several limitations must be acknowledged. First, this is a single-centre observational study and there may have been many potential confounders. Second, some of the negative results could be explained by type II error (insufficient statistical power) due to the relatively small sample size. Third, results cannot be applied to patients with LVEF <50%. Fourth, although the study sample is fairly representative of the majority of the HFpEF population, our results may not be applied to younger and milder HFpEF patients with less comorbidity burden [42]. Fifth, the use of a bicycle exercise protocol rather than treadmill exercise testing could have led to underestimation of functional capacity in some patients. Sixth, we do not have information regarding urinary albumin or protein levels, which would have allowed a more precise definition of renal dysfunction. Finally, current findings require validation in larger studies that include a wider spectrum of patients with HFpEF.

## CONCLUSIONS

In this cohort of patients with a high prevalence of advanced HFpEF, NT-proBNP was not associated with peak VO_2_ when CKD was present. CA125 emerged as a useful biomarker for estimating effort intolerance in HFpEF irrespective of the presence of CKD.

## STATEMENT OF ETHICS

This study protocol was reviewed and approved by local ethics committee. Written informed consent was obtained from all participants.

## Supplementary Material

sfae199_Supplemental_File

## Data Availability

The data that support the findings of this study are not openly available but are available from the corresponding author upon reasonable request.
