# Supplementary material for: CA125 outperforms NT-proBNP in the prediction of maximum aerobic capacity in heart failure with preserved ejection fraction and kidney dysfunction
Source: Clin Kidney J. 2024 Jul 2;17(8):sfae199. doi: 10.1093/ckj/sfae199 (PMC11317843; doi:10.1093/ckj/sfae199)
Supplement: sfae199_Supplemental_File [file sfae199_supplemental_file.docx]

**Supplementary material**

**Supplementary table 1.** STROBE (Strengthening The Reporting of Observational Studies in Epidemiology) checklist.

|  | Item No | Recommendation | Page No |
| --- | --- | --- | --- |
| **Title and abstract** | 1 | (*a*) Indicate the study’s design with a commonly used term in the title or the abstract | 3 |
|  |  | (*b*) Provide in the abstract an informative and balanced summary of what was done and what was found | 3 |
| Introduction | | |  |
| Background/rationale | 2 | Explain the scientific background and rationale for the investigation being reported | 6 |
| Objectives | 3 | State specific objectives, including any prespecified hypotheses | 7 |
| Methods | | |  |
| Study design | 4 | Present key elements of study design early in the paper | 7 |
| Setting | 5 | Describe the setting, locations, and relevant dates, including periods of recruitment, exposure, follow-up, and data collection | 7 |
| Participants | 6 | (*a*) *Cohort study*—Give the eligibility criteria, and the sources and methods of selection of participants. Describe methods of follow-up  *Case-control study*—Give the eligibility criteria, and the sources and methods of case ascertainment and control selection. Give the rationale for the choice of cases and controls  *Cross-sectional study*—Give the eligibility criteria, and the sources and methods of selection of participants | 7 |
|  |  | (*b*) *Cohort study*—For matched studies, give matching criteria and number of exposed and unexposed  *Case-control study*—For matched studies, give matching criteria and the number of controls per case |  |
| Variables | 7 | Clearly define all outcomes, exposures, predictors, potential confounders, and effect modifiers. Give diagnostic criteria, if applicable | 8 |
| Data sources/ measurement | 8* | For each variable of interest, give sources of data and details of methods of assessment (measurement). Describe comparability of assessment methods if there is more than one group | 8, 9 |
| Bias | 9 | Describe any efforts to address potential sources of bias | 8, 9 |
| Study size | 10 | Explain how the study size was arrived at. | / |
| Quantitative variables | 11 | Explain how quantitative variables were handled in the analyses. If applicable, describe which groupings were chosen and why | 8, 9 |
| Statistical methods | 12 | (*a*) Describe all statistical methods, including those used to control for confounding | 8, 9 |
|  |  | (*b*) Describe any methods used to examine subgroups and interactions | 8, 9 |
|  |  | (*c*) Explain how missing data were addressed | / |
|  |  | (*d*) *Cohort study*—If applicable, explain how loss to follow-up was addressed  *Case-control study*—If applicable, explain how matching of cases and controls was addressed  *Cross-sectional study*—If applicable, describe analytical methods taking account of sampling strategy | / |
|  |  | (*e*) Describe any sensitivity analyses | / |

**Supplementary table 2.** Associations between circulating biomarkers (NT-proBNP and CA125) and peakVO2 in ambulatory HFpEF across eGFR status. Uni and multivariates estimates.

|  | **β-coefficient (CI 95%)**  **mL/kg/min** | **β-coefficient (CI 95%)**  **mL/kg/min** |  |
| --- | --- | --- | --- |
|  | **eGFR≥60 mL/min/1.73m2** | **eGFR<60 mL/min/1.73m2** | **p-value for interaction** |
| **NT-proBNP (per increase in 1000 pg/mL)** | | | |
| Univariate | -0.85 ( -1.61 to -0.09) | -0.30 (-0.56 to -0.5) | 0.167 |
| Model 1 | -1.18 (-1.93 to -0.45) | -0.24 (-0.49 to -0.01) | 0.019 |
| Model 2 | -0.59 (-1.23 to -0.04) | 0.02 (-0.19 to 0.23) | 0.045 |
| **CA125 (per increase in 5 U/mL)** | | | |
| Univariate | -0.16 (-0.27 to -0.04) | -0.12 (-0.20 to 0.01) | 0.413 |
| Model 1 | -0.17 (-0.29 to -0.05) | -0.15 (-0.24 to -0.04) | 0.574 |
| Model 2 | -0.18 (-0.34 to -0.02) | -0.19 ( -0.35 to -0.02) | 0.620 |

Model 1: adjusted for age and sex.

Model 2: adjusted for age, sex, hemoglobin, body mass index (BMI), Charlson comorbidity index, atrial fibrillation, chronotropic index, RER, left ventricular ejection fraction (LVEF), indexed left ventricular volumes, and E/e´.
